# Editorial: Intuitive eating, health, and body: research, prevention, and treatment

**DOI:** 10.3389/fnut.2024.1406345

**Published:** 2024-05-01

**Authors:** Mona Vintila, Kamila Czepczor-Bernat, Petra Rust

**Affiliations:** ^1^Clinical Psychology and Psychotherapy, Department of Psychology, West University of Timişoara, Timişoara, Romania; ^2^Department of Pediatrics, Pediatric Obesity and Metabolic Bone Diseases, Faculty of Medical Sciences in Katowice, Medical University of Silesia, Katowice, Poland; ^3^Department of Nutritional Sciences, Faculty of Life Sciences, University of Vienna, Vienna, Austria

**Keywords:** intuitive eating, health, body image, nutritional education, eating behavior

In both clinical and non-clinical populations (such as those with overweight or obesity, patients with binge eating disorders, or those with chronic diseases like type 2 diabetes mellitus), intuitive eating has been recognized for its important role in promoting healthy eating behavior. Knowledge in this field is constantly expanding, with authors providing new and fascinating findings. In this Research Topic, our intention was to focus on the latest discoveries regarding the relationship between intuitive eating, health, and body/body image.

In the paper *Association between soybean product consumption and executive function in Chinese Tibetan children and adolescent* (Yin et al.) it was found that with the continuous decline of soybean product consumption, the subjects' refreshing memory function and conversion flexibility showed a tendency to decrease. Maintaining appropriate soybean product intake has an important role and significance in the development of brain executive function in Tibetan children and adolescents at high altitude. The results of the present study confirm that there is an association between soybean product consumption and inhibitory control and translational flexibility of brain executive functions in Tibetan children and adolescents at high altitude in China, and that decreased soybean product consumption is associated with an increased risk of developing inhibitory control and translational flexibility dysfunctions.

*Intuitive eating has reduced the chances of being overweight in university students during the COVID-19 pandemic* (Souto et al.). University students have been particularly affected during the COVID-19 pandemic, and several sociodemographic and behavioral factors may be associated with the risk of overweight in this population. There was an increase in BMI and intuitive eating score during the pandemic. This study showed that sociodemographic, lifestyle and behavioral variables had both a positive and negative influence on nutritional status. Intuitive eating was shown to be a protective factor during this period, reducing the chances of being overweight in this population. Thus, supporting intuitive eating may favor greater weight stability, and can therefore have helped to reduce the impact of the pandemic on weight gain. In this way, people who eat more intuitively partially resisted the context that favored weight gain (stress, changes in nutritional behavior and physical inactivity).

*Association between body weight perception and intuitive eating among undergraduate students in China: the mediating role of body image* (Zhu et al.). The current findings demonstrated that in addition to the direct effect of body image on intuitive eating, body weight perception also affected intuitive eating. Intuitive eating did not greatly change with age, suggesting that its overall trend is relatively stable. Notably, there was a difference between the actual body weight and the body weight perception, and students were prone to perceive themselves as overweight. The authors corroborated a significant indirect relationship between body weight perception and intuitive eating in both the normal weight and overweight groups. Regardless of how accurately the students estimated their weight, the heavier they subjectively perceived themselves to be, the more negative they were about their body image. According to the study results, a realistic assessment of body weight is essential to facilitate intuitive eating.

*Nutrition education and its relationship to body image and food intake in Asian young and adolescents: A systematic review* (Shivappa Pushpa et al.). This study aimed to conduct a systematic review of the literature on the effect of nutritional education interventions on nutritional knowledge and food intake behavior, attitude, practice, and body image in children and adolescents. Most of the nutritional education interventions lowered unhealthy food intake and body image misperception, particularly by enhancing nutritional knowledge/self-efficacy, healthy dietary habits, physical activities, and fruit and vegetable intake.

*My Diet Study: protocol for a two-part observational, longitudinal, psycho-biological study of dieting in Australian youth* (Okada et al.). This paper described the protocol for the My Diet Study, a two-arm observational investigation of the natural progression of dieting among young people over a period of 6-months. The study aims were to examine the links between self-directed dieting, general physiological and psychological metrics of wellbeing (e.g., depressive symptoms) and biomarkers of gut-brain axis functions (e.g., microbiome and hormones) that are predicted to influence diet adherence through appetite, mood and metabolism regulation. The results may provide potential biopsychosocial solutions (e.g. psychoeducation on the importance of diet for a health-promoting microbiota) to improve safety in dieting among young people and help to establish the potential for biomarkers for risk management and improvement of diet-based lifestyle interventions.

In the pursuit of intuitive eating, it's imperative to recognize the intricate interplay between nutrition, psychology, and body perception.

Nutrition forms the basis for physical wellbeing, providing essential nutrients for proper body functions. A balanced diet not only provides the body with energy, but also influences mood, cognition, and behavior.

Equally important is the role of psychology in shaping eating habits and lifestyle choices. Our relationship with food is complex and is often influenced by emotions, stress, and social norms. Emotional eating, for example, can lead to unhealthy eating patterns that are driven by convenience rather than nutritional necessity.

In addition, body perception plays an important role in shaping attitudes toward body weight and health. In a society inundated with unrealistic beauty standards, a distorted body image can lead to disordered eating behavior, and a constant cycle of dissatisfaction.

To summaries, achieving intuitive eating requires a comprehensive understanding of the complex interplay between nutrition, psychology, and body perception. By promoting both physical and mental wellbeing, we can help cultivate a healthy relationship with food, support a positive body image and ultimately empower people to make sustainable lifestyle changes for long-term health.

## Author contributions

MV: Conceptualization, Writing – original draft, Writing – review & editing. KC-B: Conceptualization, Writing – original draft, Writing – review & editing. PR: Conceptualization, Writing – original draft, Writing – review & editing.

